# Impact of automobile exhaust on biochemical and genomorphic characteristics of *Mimusops elengi* L. growing along roadsides of Lahore city, Pakistan

**DOI:** 10.1016/j.heliyon.2024.e28157

**Published:** 2024-03-14

**Authors:** Ali Hasnain, Hamed Dadkhah-Aghdash, Muhammad Luqman, Sohaib Muhammad, Andleeb Anwar Sardar, Shaukat Ali, Farhat Mehmood, Usman Ahmed Khan, Zahid Mehmood, Arooba John, Binyameen, Zafar Iqbal Khan, Hsi-Hsien Yang, Muhammad Umer Farooq Awan

**Affiliations:** a Department of Botany, Government College University, Lahore, 54000, Pakistan; b Department of Plant Biology, Faculty of Biological Sciences, Tarbiat Modares University, Tehran, Iran; c Department of Environmental Sciences, University of Veterinary and Animal Sciences (UVAS), Lahore, Pakistan; d Department of Zoology, Government College University, Lahore, 54000, Pakistan; e Institute of Molecular Biology and Biotechnology, University of Lahore, Lahore, 54000, Pakistan; f Department of Botany, University of Sargodha, Sargodha, Pakistan; g Department of Environmental Engineering and Management, Chaoyang University of Technology, Taichung, 413310, Taiwan

**Keywords:** Vehicular pollution, Air pollution tolerance, Comet score, DNA damage, Heavy metals, Lahore city

## Abstract

Automobile exhaust releases different types of pollutants that are at great risk to the air quality of the environment and incidental distress to the nature of roadside plants. *Mimusops elengi* L. is an evergreen medicinal tree cultivated along the roadside of Lahore City. This research aimed to investigate physiological, morphological and genomorphic characteristics of *M. elengi* under the influence of air pollution from vehicles. Healthy and mature leaves were collected from trees on Canal Bank and Mall roads of Lahore as the experimental sites and control sites were 20 km away from the experimental site. Different physiochemical, morphological, air pollution tolerance index (APTI) and molecular analysis for the detection of DNA damage were performed through comet assay. The results demonstrated the mean accumulated Cd, Pb, Cu and Ni heavy metal contents on the leaves were higher than the control plants (1.27, 3.22, 1.32 and 1.46 μg mg^−1^). APTI of trees was 9.04. Trees in these roads significantly (p < 0.01) had a lower leaf area, petiole length and leaf dry matter content in comparison to control site. Increased comet tail showed that DNA damage was higher for roadside trees than trees in the control area. For tolerance of air pollution, it necessary to check the APTI value for the *M. elengi* at the polluted road side of Lahore city. For long-term screening, the source and type of pollutants and consistent monitoring of various responses given by the trees should be known.

## 1 Introduction

Air is the most fundamental resource for maintaining life on the earth, all living organisms rely on clean air for optimal development and growth in their natural habitat. Rapid industrialization and urban development have significantly increased the air pollution level in urban areas. The transport division is a key source of air pollution [1]. Vehicular exhaust affects the health of people and vegetation which settles in the cities [2]. Plants play an essential role in all types of ecosystems and are highly susceptible to the effects of air pollutants. Plants, being immovable, are persistently subjected to the chemical pollutants present in the environment [1]. The green urban areas are crucial for facilitating social interactions and providing a peaceful environment that promotes inner harmony and well-being. Urban green areas can be a possible strategy to reduce the negative impacts of urbanisation on the environment. Lahore city ranks second in size and serves as the provincial capital of Punjab, Pakistan. The city is alternatively known as the “City of Gardens” and is regarded as a prominent centre for education, culture, politics, economy, transportation and entertainment [3]. Automobile exhaust is a major contributor to substantial air pollution in Lahore city. Numerous investigations revealed a strong correlation between road traffic and particulate matter (PM). The significant traffic on the roads leads to the release of air pollutants into the environment.

The flow of traffic in Lahore has significantly impacted the air quality of the city by generating aerosols that include a substantial concentration of various heavy metals like Cd, Zn and Pb. They are released into the environment and affect the physiological properties of plant species [4]. The increased traffic and pollution in Lahore after the year 2000 are the results of a spike in the city's automobile population [5]. Lahore is one of the most polluted cities in the world while unplanned urbanization and industrialization are also contributing to polluting the air of Lahore. Roadside plants are the first targets of vehicular exhaust. Plants for tolerance of atmospheric pollution in cities especially exhausted emissions change their morphological, biochemical and physiological characteristics [6].

*Mimusops elengi* (*M. elengi*) L. is distributed in the tropical and subtropical areas of Asia. This woody ornamental plant can reach a height of 15 m. The tree is an evergreen species characterised by a smooth, large glabrous trunk and fragrant blossoms. It is indigenous to southern India, Burma, and Pakistan. Plants that are growing in air-polluted areas have reactive oxygen species (ROS) to cope with stress [7]. This tree is renowned for its ample shade and its graceful appearance. It is advisable to increase the cultivation of *M. elengi* [8]. Furthermore, it primarily serves as a medicinal tree for treating wounds, pain, inflammation, antinociceptive effects, diuretic effects, gastroprotective properties, antibacterial and antifungal properties, free radical scavenging abilities etc. The leaves ethanolic extract contained quercitol, hentriacontane, and carotene in addition to glucose. The compounds D-mannitol, sitosterol, sitosterol-D-glucoside and quercetin can be isolated from the leaves [9]. Muhammad et al. [10] measured the air pollution tolerance index (APTI) for the four tree species at Mall and Jail roads of Lahore city and found that various results were sensitive and tolerant to air pollution. APTI is a critical parameter that is applied to detect the tolerance capability of different roadside plants which is based on their biochemical constituents such as relative water content chlorophyll contents, ascorbic acid corrosive and pH values.

Potential sources of air pollution released PM and the subsequent production of ROS. Oxidative stress caused by exposures triggers the activation of inflammatory pathways, including mitogen-activated protein kinase, nuclear factor-kappa B and activator protein 1, resulting in elevated cytokine levels [11]. Assessing the genotoxic impact of pollutants on plants is critical because plants play a significant role in environment and serve as a fundamental component in the food chain [12]. Pollutants emitted from vehicle emission have potential to caused DNA damage. In the field of ecotoxicology, many plants are employed to assess genetic damage [13]. Plants, being constantly exposed to a combination of contaminants, are acknowledged as bio-indicators of DNA destruction resulting from urban atmosphere stresses. Consequently, diverse sections of higher plants are commonly employed for air pollution biomonitoring [14].

Plants used as bio-accumulated because they absorb the different trace elements such as Zn, Cu, Pb and Cd but its extreme concentration is dangerous for plant health [15]. Due to its lengthy half-life, Pb concentrations are anticipated to be elevated in potential environmental contamination [16]. Cd is a potentially lethal metal for the environment due to its toxicity to both humans and other animals. Electronic waste and anthropogenic corridors are the main sources of Cd. Human health is affected in several ways by these harmful heavy metals particularly Cd [17]. Heavy metals have a detrimental impact on the growth and advancement of plants also affected their development. Plant species vary in their capacity to assimilate significant amounts of heavy metals. The gathering of key metals and the metal magnitude relationship are influenced by the morph-physiological properties of plants.

Previous studies have demonstrated a decline in the levels of heavy metals in leaves of plant far away from the road. Results demonstrated that road traffic emissions are responsible for these pollutants, including heavy metals and their connection with APTI. Plants with higher APTI scores exhibit higher tolerance to air pollution. The APTI of trees is affected by natural factors such as ascorbic acid, total chlorophyll, pH and water content. The level to which plants take up heavy metals is correlated with their biochemical features, as indicated by the APTI values of the trees. When APTI values rise, the level of Cd absorption by plants also increases. There exists a positive correlation between APTI and the absorption of heavy metals by trees, as in the case of Pb and Ni. However, the correlation between Zn and APTI was determined to be comparatively weaker. This indicates that there is a minimal correlation between APTI and Zn [18]. The study reported by Villarini et al. [19] revealed a strong positive association between the concentrations of NO and PM_10_ and the level of DNA damage in *Nicotiana tabacum*. The modern urban development strategy could help reduce the environmental impacts of urbanisation. The selection of trees with high biomass or a wide canopy cover with fast urbanisation in mind to achieve the greatest possible carbon mitigation. Some roads in Lahore city have large traffic flow such as Mall road and Canal Bank road, due to most of the offices and colleges are present on these roads.

This research aims to investigate the heavy metals (Cu, Pb, Cd and Ni) absorption by leaf, physiochemical and morphological parameters and the genotoxicity caused by roadside air pollution. Trees located at highly polluted sites used different mechanism for tolerance against air pollution with respect to less polluted site. In this regard, this research evaluated the morphological and physiochemical parameters for *M. elengi* tree and molecular approach for a decision to use that specific specie for the sustainable environmental goals in Lahore city.

## 2 Materials and methods

### 2.1 Study areas and site selection

**Fig. 1:**
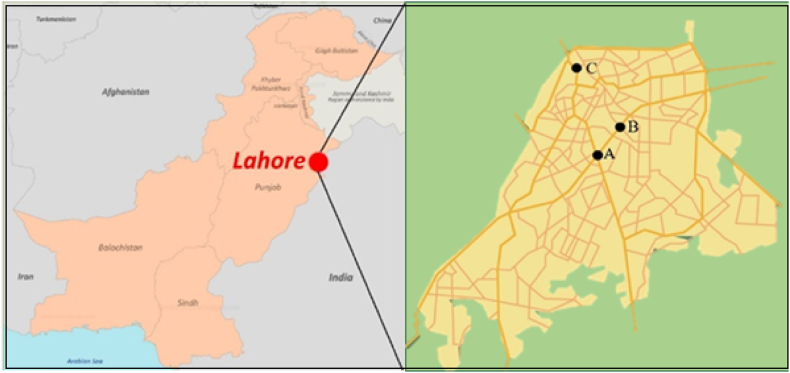
Map of study areas. A: Canal Bank road (Moderate traffic area) B: Mall road (Intensive traffic area) C: Control site.

**Fig. 2:**
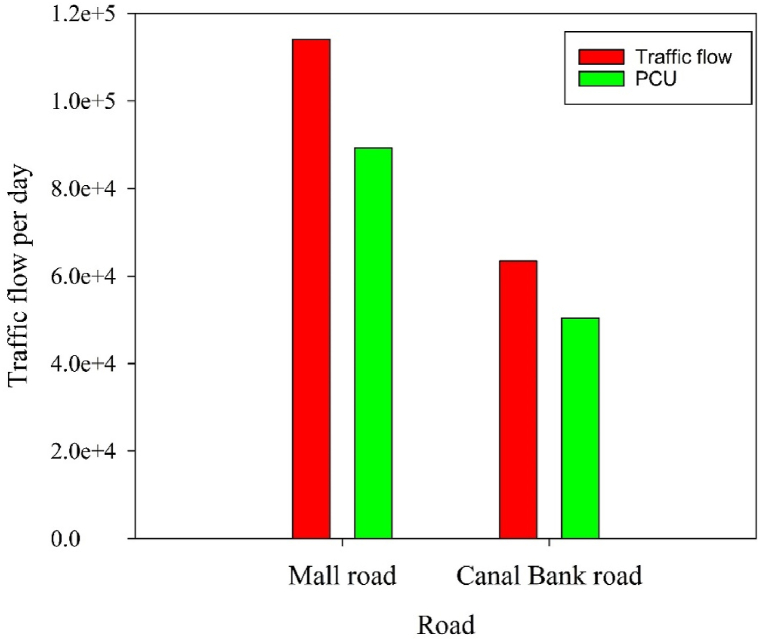
Traffic flow on the Mall road (intensive traffic area) and Canal Bank road (moderate traffic area).

Lahore city is situated in the Punjab province of Pakistan and is positioned between 74° 20′ 37″ E and 31° 32′ 59″N latitude and longitude correspondingly cover a region of 1772 km. The atmosphere of the investigated area is semi-arid, the hottest months are June and July and the temperature ranges between 40 and 48 °C, and December and January are coldest months [4]. The busiest roads in the Lahore metropolitan area are Mall Road and Canal Bank road, where heavy traffic was observed every day. Twelve underpasses on Canal Bank road enable constant traffic movement from Thokar Niaz Baig to Harbanspura. Due to the presence of significant private-public offices, businesses and commercial marketplaces along Mall Road, there has been an increase in traffic flow as compared to Canal Bank road. The passenger car unit (PCU) is used to calculate the heterogeneity volume of traffic. PCU for Mall road is higher as compared to Canal Bank road [20]. Two main roads were selected as experimental sites and the control site (20 km away from Mall and Canal Bank road) (Fig. 1). According to Lahore Traffic Engineering and Planning Agency, the total number of vehicles flowing through Mall Road and Canal Bank road in all directions was 116,125 and 64,197 day^−1^, respectively (Fig. 2). The average air quality index (AQI) values taken by Environment Protection Agency for the Mall road and Canal Bank road were 230 and 183 while for control site was 78 during the sampling period of this study.

### 2.2 Plant sampling

The *M. elengi* leaf samples were collected between April 2022 and September 2022 following method of [21]. Random 10 fresh matured leaves were gathered from 2-m height of different trees located around roadside. The identification of *M. elengi* was completed at Sultan Ahmed Herbarium, Government College University Lahore, Pakistan. *M. elengi* was selected because of their presence at both control and experimental sites. The tree species chosen had almost equal trunk diameter and age. Leaf samples were transferred to laboratory in a handy icebox for physiological study. Samples were rinsed and assessed on the same day and were stored in a refrigerator for further experiment.

### 2.3 Heavy metal analysis of leaf samples

The leaf samples were dried in placing at 70 °C oven. The leaves were pulverised into an adequate powder operating a sterile electric grinder. Take 2 g sample of powdered leaves in a Pyrex beaker adding 10 mL of pure nitric acid. The mixture was left at room temperature overnight. Subsequently, the sample was heated gently, it was then cooled, and 5 mL of HClO_4_ was introduced. The digested samples were filtered into a volumetric flask with the addition of ddH_2_O, and the volume was raised up to 50 mL. The extracts were analysed for heavy metals including Cd, Ni, Pb and Zn through atomic absorption spectrophotometer (Thermo Fisher Scientific iCE 3000 Series’ AAS). The certified standard reference materials NCS DC 73350 Poplar were used for the verification of this method.

### 2.4 Assessment of morphological, physiochemical and molecular parameters

#### 2.4.1 Assessment of morphological parameters

For the measurement of morphological parameters, 10 healthy and mature leaves were selected from each tree. Leaf area was measured based on the grid counting method. Fresh and dry weight (after 70 °C for 15 h in an oven) of leaf samples were measured in gram by a Hanna Weight scale (model number DH-V220A and Power DC9V) with an accuracy of 0.001 g and the length of petioles was measured by a ruler per centimetre. Specific leaf area (SLA) was calculated by dividing leaf area by leaf dry weight. Leaf dry matter content (LDMC) was analysed from the ratio of leaf dry weight to fresh weight.

#### 2.4.2 Assessment of physiochemical parameters

For the physiochemical assessments leaf samples with comparable shapes and ages were chosen. Extract of leaves was prepared with 80% (v/v) acetone and centrifuged. The ratio of light absorbance was estimated at 645 nm and 663 nm on a UV–visible spectrophotometer (UV752, D model). Calculation of chlorophylls was made by the following Eq. (1) [22].

(1) $${\text{Total}\;\text{Chlorophyll}\;b\;(g\;L}^{-1})=\left(0.0202\right)\;\left(A645\right))\left(0.00802\right)\;\left(A663\right)$$

where,

A663 and A645 refer to the absorbance at 663 and 645 nm, respectively. Chlorophyll content was expressed according to the extract amount and sample weight established on milligrams per gram of fresh weight (mg g^−1^ FW). Relative water contents (RWC) of the selected leaves were measured by using Eq. (2) [23].

(2) $$\text{RWC}\;\left(\%\right)=\frac{\left(\text{FW}-\text{DW}\right)}{\left(\text{TW}-\text{DW}\right)}\times 100$$

where,

RWC is relative water content (%), FW is fresh weight (g) while DW is dry weight (g) of leaves after drying the leaves in a hot incubator and TW is the turgid weight of leaves (g) obtained by immersion in distilled water overnight. For the determination of pH 2 g fresh leaves were homogenized in 50 mL dH_2_O and centrifuged at 2000 rpm for 10 min. The supernatant pH was measured with the help of a digital pH meter. While the concentration of ascorbic acid was measured by the redox titration method. Sample solution 2 mL was taken in 250 mL conical flask. Fifty mL of distilled water (50:1 v/v) and 2 mL of 0.5% starch indicator solution were added. The sample was then titrated with an Iodine solution. Initial and final readings were calculated by observing the blue-black color as an endpoint. The air pollution tolerance index of tree species was calculated by using the following Eq. (3) [24].

(3) $$\text{APTI}=\frac{\left[A\;\left(T+P\right)\right]+R\;}{10}$$

where,

A = Ascorbic acid concentration (mg g^−1^)

T = Chlorophyll content (mg g^−1^)

P = leaf extract pH.

R = Relative water contents of the leaves (%)

#### 2.4.3 Molecular analysis

##### 2.4.3.1 Genomic DNA extraction, gel electrophoresis and DNA quantifications

The genomic DNA was extracted by using the CTAB method with little modifications. Agarose gel electrophoresis was performed to check the quality, size and presence of DNA by running on 1% agarose gel Using a UV–visible Spectrophotometer-2373, DNA was quantified after agarose gel electrophoresis.

##### 2.4.3.2 Comet assay

Comet assay technique was used to assess the genotoxic effect of vehicle exhaust on the DNA of roadside trees and compared with the control DNA located at less polluted site. Comet assay or single-cell gel electrophoresis is a simple, versatile and sensitive method that can be used to detect DNA damage in the cell. Cellular comet assay illustrates the DNA-damaging effect of air pollution where concentration of heavy metals is higher [25,26]. A neutral comet assay was used to check the breakdown of the double-stranded DNA by the method described by Ref. [27].

##### 2.4.3.3 Visualization of DNA damage through comet assay IV software

The breakage of DNA was visualized at a 10× objective lens by placing the prepared slides under a fluorescence microscope (Olympus BX50, Hamburg, Germany). Images were taken with a high-resolution camera. Comet Assay IV software was applied to check the head-tail migration.

### 2.5 Statistical analysis

The concentration of Cd, Pb, Cu and Ni in leaves, as well as biochemical, physiological and morphological parameters of leaves with three replications were examined in a completely randomized design. Analysis of variance by one-way ANOVA and a similarity of the means of all measured parameters was concluded using the Duncan test at p < 0.01 by SPSS software version 24. Different quantity DNA measurements were done based on SPSS data analysis.

## 3 Results

### 3.1 Heavy metal concentration in leaf samples

**Fig. 3:**
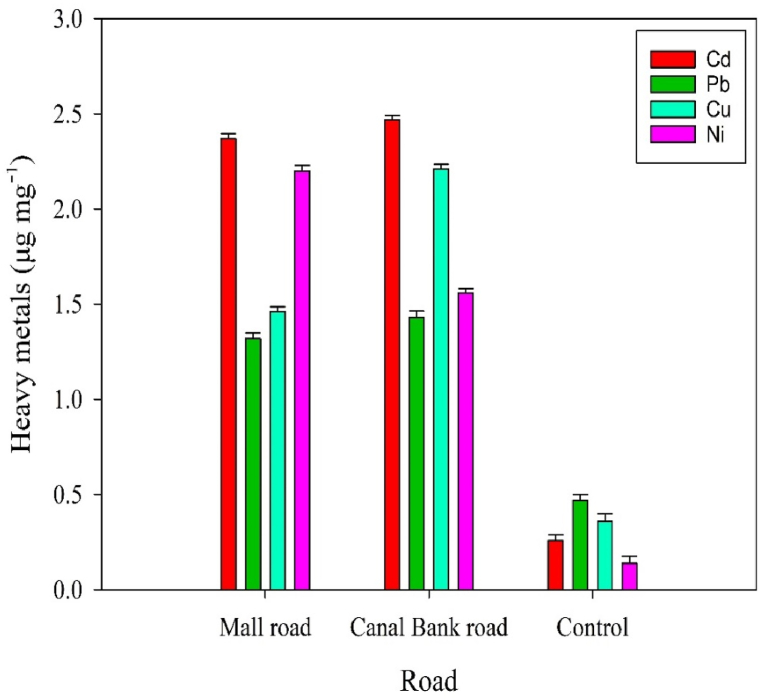
The concentration of heavy metals in the leaf samples of *M. elengi* at the different roads.

The results of heavy metals analysis of leaf samples showed that the Cd concentration of Canal and Mall roads was higher than the control site (2.47 ± 0.0205, 2.37 ± 0.0249 and 0.26 ± 0.0264 μg mg^−1^). Leaf samples of Pb for Canal and Mall roads had heavy metals 1.43 ± 0.0326 and 1.32 ± 0.0286 μg mg^−1^ Pb, but the control site had the lowest content (0.47 ± 0.0305 μg mg^−1^). The Cu concentration in leaves with 2.2 ± 0.0249 and 1.46 ± 0.0249 μg mg^−1^ in these roads was higher than control site trees (0.36 ± 0.0360 μg mg^−1^). Ni concentration of leaves for these roads were 1.56 ± 0.0205 ± μg mg^−1^ and in the control site was 0.14 ± 0.0351 μg mg^−1^ (Fig. 3).

**Fig. 4:**
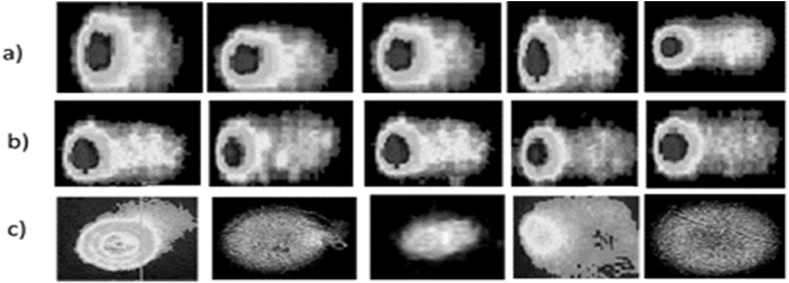
Interpretation of comet assay by using the Comet Assay IV software. (a) Canal Bank road, (b) Mall road, (c) Control site.

### 3.2 Morphological parameters

The results of the leaf morphological parameters assessment showed that the leaf area of Canal Bank and Mall roads trees was significantly (p < 0.01) lower than control site trees. In addition, trees of these roads significantly had lower petiole length and specific leaf area (2.39 cm and 14.93 cm^2^ g^−1^) as compared to the control site. LDMC of trees of Canal Bank road against Mall Road had no significant difference (p > 0.05) with control site trees (Table 1).

**Table 1:** The effect of air pollution on some leaf morphological parameters.

Site	Leaf area (cm^2^)	Petiole length (cm)	Specific leaf area (cm^2^ g^−1^)	LDMC
Canal Bank road	5.83^b^ ± 0.14	1.99^b^ ± 0.07	12.71^b^ ± 2.34	12.7^ab^ ± 0.63
Mall road	5.33^b^ ± 0.11	1.95^b^ ± 0.04	11.85^b^ ± 1.35	11.84^b^ ± 0.33
Control site	7.87^a^ ± 0.45	2.39^a^ ± 0.02	14.93^a^ ± 1.79	14.92^a^ ± 0.17

According to the Duncan test, the means with different letters are significantly different (p < 0.01).

### 3.3 Physiochemical parameters

The results of biochemical and physiological assessments showed that the total chlorophyll content of trees in the Canal Bank and Mall roads was pointedly (p < 0.01) lower (0.37) than control site trees (0.44 mg g^−1^). RWC of trees in Canal Bank and Mall roads with 85.41% and 82.71% were significantly more than Control site trees (62.44%). The trees on these roads had higher pH in comparison with the control site. The trees of Mall Road and control had the highest and lowest ascorbic acid content (3.05 and 0.73 mg g^−1^). In addition, these trees had higher ascorbic acid content than the control site. Regarding APTI, control place trees had the lowest value (6.51) among the studied sites (Table 2).

**Table 2:** APTI of trees in different regions of Lahore.

Site	Total chlorophyll (mg g^−1^)	RWC (%)	pH	Ascorbic acid (mg g^−1^)	APTI
Canal Bank road	0.39^a^ ± 0.009	85.41^a^ ± 0.46	6.66^a^ ± 0.06	2.30^b^ ± 0.13	10.16
Mall road	0.36^a^ ± 0.008	82.71^a^ ± 0.80	6.10^a^ ± 0.05	3.05^a^ ± 0.03	10.24
Control site	0.44^b^ ± 0.030	62.44^b^ ± 1.72	3.35^b^ ± 0.14	0.73^c^ ± 0.04	6.52

According to the Duncan test, the means with different letters are significantly different (p < 0.01).

### 3.4 Molecular parameters

#### 3.4.1 Genomic DNA extraction and agarose gel electrophoresis

Genomic DNA was extracted from all samples (3 trees from each selected site). All the samples showed good-quality of genomic DNA for further experimentation.

#### 3.4.2 DNA quantification

Quantification of genomic DNA was performed to check the quality of DNA. The readings were obtained by taking the mean absorbance ratio of all samples at A260/A280 nm. The A260/A280 ratio was used as the purity indicator for DNA sample. The optimum value for A260/A280 nm ratio for pure DNA is 1.8. The 3 samples collected from the control site showed a high concentration ratio (1.89, 1.81 and 1.77) of DNA. The 3 Mall road samples showed concentrations of 2.11, 1.99 and 1.70. The lowest concentration of DNA was observed in the 3 samples of Canal Bank road (2.02, 1.98 and 2.12, respectively).

#### 3.4.3 Visualization of DNA damage through comet assay IV software

For checking the DNA damage, images of all comet slides were taken after visualization under a fluorescence microscope. After applying the images in Comet Assay IV software, mean tail length, mean comet head length, comet tail migration and total area were measured. The effect of automobile exhaust on the genome of the plants present on experimental sites is shown in Fig. [4 (a-c)]. The control site represents the negative effect. The most DNA damage was recorded on Canal Bank road rather than Mall road and the control site. There were considerable differentiations in the mean head length of plant samples in the experimental sites and control areas. Mean head length in Canal Bank and Mall roads were respectively 31.73 ± 0.64 and 11.23 ± 0.18 μm. It was 22.45 ± 0.35 μm on the control site. The highest mean tail length was observed on Canal Bank road (104.94 ± 2.01 μm), then Mall Road (80.05 ± 1.23 μm) and the control site (22.45 ± 0.35 μm). The mean tail migration of samples at the Canal Bank and Mall roads were 89.08 ± 1.28 and 74.43 ± 1.17 μm and at the control site was 0. The total area covered by the comet in Canal Bank and Mall roads samples were 44.94 ± 0.98 and 34.59 ± 0.85 mm^2^. On the other side, the lowest total area (12.31 ± 0.24 mm^2^) was observed in the control samples (Table 3).

**Table 3:** Measurement of DNA damage analysed by the Comet assay.

Site	Head length (μm)	Tail length (μm)	Tail migration (μm)	Total area (mm^2^)
Canal Bank road	31.73 ± 0.64	104.94 ± 2.01	89.08 ± 1.28	44.94 ± 0.98
Mall road	11.23 ± 0.18	80.05 ± 1.23	74.43 ± 1.17	34.59 ± 0.85
Control site	22.45 ± 0.35	52.96 ± 1.05	0	12.31 ± 0.24

## 4 Discussion

Lahore is classified as the second highest polluted city in the world in respect of PM_2.5_ and PM_10_ [9]. The roadside trees are directly exposed to automobile exhaust and absorb the heavy metals in their organs through stomata by increasing the traffic flow and air pollution in Lahore. According to Ref. [28], the normal concentrations of Cd, Pb, Cu and Ni elements in plants are 0.05–2, 0.5–10 3–30 and 0.1–5 mg kg^−1^. Our results showed that these elements in *M. elengi* had higher contents than normal ranges for plant species. Heavy metals such as Zn, Pb, Ni and Mn were significantly accumulated in the leaf organs of *Robinia pseudoacacia* L. of Denizli City, Turkey [29]. Air pollution is among environmental stresses that threaten the well-being of people as well as the lives of plant species in urban ecosystems, especially roadside trees. These trees with high cover and absorption rate of air pollution give different reactions and responses to the stresses.

Plants' pollution tolerance capabilities vary with species and environmental conditions [30]. Chlorophyll has a critical function in the plant physiology and is a main indicator of environmental stresses with unjustified role to play in regulating photosynthesis [31]. Chlorophylls diminish the production of ROS in the chloroplast under stress conditions that cause damage to cellular bodies and plants with high chlorophyll contents combat air pollutants. Chlorophyll contents were greater in plants in polluted areas this great content may help them to tolerate air pollutants. Higher and lower content of chlorophyll content of plant species during air pollution showed their tolerance and sensitivity to them [32]. In this research, chlorophyll content concentration for *M. elengi* plant decreased under the air polluted stress.

The increase of RWC in plants causes the removal of air pollutants as well as the increase in photosynthesis and help to cope with dry conditions. Thus, plants with high RWC are more tolerant to air pollution and helps to maintain their normal function and physiological balance regarding to tolerance air pollution [33]. *M. elengi* presents on the Canal Bank and Mall roads under air pollution stress had higher RWC that provide higher tolerance ability in these trees to this stress. Previous study concluded that the RWC of *Ficus religiosa* L. in the air-polluted areas was higher than the control site [34]. It was due to the maintenance of RWC by plants for their tolerance to air pollution.

Acidic gaseous pollutants produced by vehicles on roads cause plant species along the roadside to be more acidic [35]. pH of leaf extracts is one of the bio-indicators of plant species under air pollution. Plants with higher pH indicate that they are tolerant to air pollution [36]. Thus, *M. elengi* with higher pH in Canal Bank and Mall roads was more tolerant to air pollution as compared to the control site. But in general, it can be said that this tree with an acidic range of pH was sensitive to urban air pollution. Ascorbic acid has an important role in diminishing reactive oxygen species and protecting the thylakoid membrane from oxidative stress damage [37]. Plants during tolerance of air pollution increase their antioxidants such as ascorbic acid for tolerance of air pollution. Trees in Canal Bank and Mall roads had higher ascorbic acid contents as compared to control site trees.

In this research, *M. elengi* had higher APTI in experimental roads as compared to the control site. In general, according to this index, this tree was sensitive to air pollution. Previous study found that the APTI of *Ficus religiosa* L. in polluted areas of the city was higher than the control site [38]. They concluded that trees with higher APTI had a high tolerance to air pollution.

Evaluation of various morphological responses of plants at the roadsides showed that leaves used defence mechanisms including decreasing their area and minimizing to exposure of air pollution regarding mitigate to air pollution. Length, width and area of the leaf are among the morphological traits of leaves that are negatively affected by air pollution. The sampled leaves of *Alstonia scholaris* L. cultivated along the polluted roads of Lahore city had a lower leaf area than the control site [28]. Petiole length is an important factor of plant leaves that mainly decreases in polluted areas in comparison to cleaned sites. A decline in leaf area and petiole length reduces exposure to environmental pollutants, especially air pollution and makes plants resistant to air pollution [39]. Leaf thickness and density are determined by using SLA. Thicker leaves of plants can absorb more light. SLA depends on the degree of shading and air pollution accumulation on the leaf surface. It is also dependent on the kind of plant species and the adaptive mechanism of plants. The decrease in SLA because of atmospheric pollution demonstrated that plants use large materials to build protective structures to diminish the adverse effects of air pollution on leaves. Plants located in urban areas with high atmospheric particle concentrations have a relatively low SLA due to long-term adaptation to urban air pollution [40]. Regarding the above information, trees in air-polluted roads of Lahore had lower SLA than control trees for adaptation and tolerance of air pollution.

The amount of LDMC in a plant determines its nutritional status, and a high quantity can direct a slower rate of nutrition production and consumption [41]. In our research, trees had unaffected and decreased LDMC as compared to control trees. It can be said that in air pollution conditions nutritional production and consumption of trees were increased for combating it.

Plants have developed a complex network of mechanisms specifically designed to detect and repair DNA damage, dedicated to ensuring their genomic stability by removing DNA damage and restoring the original genetic information [42]. Single-gel electrophoresis or comet analysis is the main and most common technique used to detect different types of DNA damage such as fragmentation, double and single-strand DNA breaks and base oxidation [43]. Cellular comet assay demonstrates the DNA-damaging effect of Pb, these results suggest that the DNA damage detection under high lead concentrations. Head length, tail length and tail migration are some comet assay parameters that have been used worldwide for the determination of DNA damage [44]. The fraction of DNA in the tail region (tail migration) has been used to quantify DNA strand breaks and is the most recommended parameter to use [45]. The values obtained through Comet Assay IV software, the tail migration, and other parameters of the comet assay showed that the plant existing on Canal Bank road has additional DNA damage than Mall road. Control site showed negative results. The roadside plants showed higher levels of DNA damage than non-roadside samples. Another study showed the same result in which DNA damage was seen in plants exposed to sites where the vehicular flow is very high, which may be related to the statement that DNA strands may be broken due to contact with free radicals, organic and inorganic contaminants, heavy metals, etc. [46]. Plants that are present in contaminated sites had higher levels of DNA fragmentation than those in uncontaminated sites. Also, more DNA contamination was observed in infected plants than in control plants [47].

## 5 Conclusion

Trees in urban ecosystems are confronted by air pollution from various emitted sources such as vehicular traffic. Bioaccumulation of Cd, Cu, Pb and Ni were found higher in leaves of *M*. *elengi* compared to the control site. The APTI value of trees along these roads was higher than the control site. Higher APTI values depict the sensitivity of *M. elengi* to air pollution. The physicochemical and morphological analysis results *M. elengi* lowered for polluted site as compared to control. These results indicated the high effect of air pollution on the plant at busiest road of Lahore city. The molecular analysis showed that DNA damage occurred due to air pollution and hence it caused genotoxicity. *M. elengi* can use as a bioindicator for air pollution mitigation like in. This research can help for future studies about categorization of sensitive pollution trees for urban greening design. Advance studies are required to determine the damage mechanisms of vehicular exhaust in trees present along the roadside. However, our results support the applicability and sensitivity of the comet assay for mapping plant DNA damage caused by atmospheric genotoxic agents.

## Additional information

No additional information is available for this paper.

## Data availability statement

Data will be made available on request.

## CRediT authorship contribution statement

**Ali Hasnain:** Formal analysis. **Hamed Dadkhah-Aghdash:** Writing – original draft, Data curation. **Muhammad Luqman:** Writing – original draft. **Sohaib Muhammad:** Writing – original draft, Software. **Andleeb Anwar Sardar:** Writing – original draft, Methodology. **Shaukat Ali:** Writing – original draft, Investigation. **Farhat Mehmood:** Software, Methodology. **Usman Ahmed Khan:** Investigation, Data curation. **Zahid Mehmood:** Writing – review & editing. **Arooba John:** Investigation. **Zafar Iqbal Khan:** Investigation, Conceptualization. **Hsi-Hsien Yang:** Writing – review & editing, Validation, Supervision. **Muhammad Umer Farooq Awan:** Writing – review & editing, Supervision, Project administration.

## Declaration of competing interest

The authors declare that they have no known competing financial interests or personal relationships that could have appeared to influence the work reported in this paper.
